# The Tumor Microenvironment and Strategies to Improve Drug Distribution

**DOI:** 10.3389/fonc.2013.00154

**Published:** 2013-06-10

**Authors:** Jasdeep K. Saggar, Man Yu, Qian Tan, Ian F. Tannock

**Affiliations:** ^1^Department of Medical Biophysics, University of Toronto, Toronto, ON, Canada; ^2^Division of Medical Oncology and Hematology, Princess Margaret Cancer Centre, Toronto, ON, Canada

**Keywords:** drug distribution, pharmacodynamic markers, tumor microenvironment, drug penetration, hypoxia-activated pro-drugs, solid tumor

## Abstract

The microenvironment within tumors is composed of a heterogeneous mixture of cells with varying levels of nutrients and oxygen. Differences in oxygen content result in survival or compensatory mechanisms within tumors that may favor a more malignant or lethal phenotype. Cells that are rapidly proliferating are richly nourished and preferentially located close to blood vessels. Chemotherapy can target and kill cells that are adjacent to the vasculature, while cells that reside farther away are often not exposed to adequate amounts of drug and may survive and repopulate following treatment. The characteristics of the tumor microenvironment can be manipulated in order to design more effective therapies. In this review, we describe important features of the tumor microenvironment and discuss strategies whereby drug distribution and activity may be improved.

## Introduction

### Solid tumors and drug resistance

#### The tumor microenvironment within solid tumors

**Figure 1:**
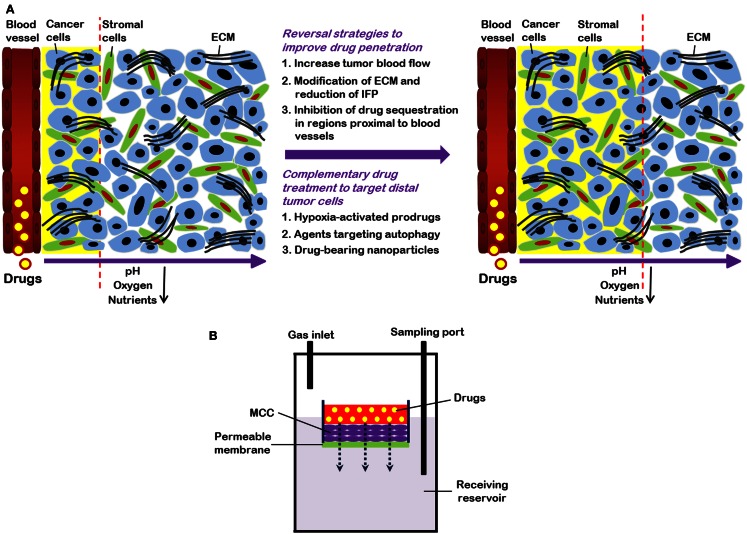
**(A)** Strategies to overcome limited drug distribution in solid tumors. Solid tumors are featured by irregular and poorly organized vasculature. This makes blood-borne oxygen and nutrients difficult to reach tumors cells distant from vessels and eventually leads to formation of regions with low oxygen (hypoxia) and nutrient concentrations. In these areas, tumor cells are usually highly resistant to chemotherapy and radiation therapy. Drug distribution in solid tumors is influenced by many factors, such as physicochemical properties of drugs, consumption of drugs by cells proximal to blood vessels, and the volume and organization of the extracellular matrix (ECM). Strategies to enhance drug distribution in tumors (indicated by yellow background and dashed lines) include increase of tumor blood flow, decrease of high interstitial fluid pressure (IFP), and modification of ECM. Combination treatment using “conventional” therapeutics together with drugs (e.g., hypoxia-activated pro-drugs and agents targeting autophagy) that are able to specifically target cells distant from vasculature also have potential to improve therapeutic efficacy. **(B)** Schematic representation of multilayered cell cultures (MCCs) to quantify drug penetration. A drug is first added into the small compartment above the MCC. After its passage from the semi-liquid media through the MCC, drug is sampled from the receiving compartment below the MCC and measured.

Solid tumors contain a heterogeneous mixture of tumor cells and non-malignant cells within an extracellular matrix (ECM) supported by an irregular vascular network. Tumor blood vessels are often farther apart than in normal tissues, and have variable blood flow, leading to poor delivery of nutrients and impaired clearance of metabolic breakdown products from the tumor (Minchinton and Tannock, 2006; Tredan et al., 2007). Many solid tumors develop regions of hypoxia, which may lead to up-regulation of genes that predispose to a more malignant phenotype (Wilson and Hay, 2011). Blood vessels are also the route by which anticancer drugs are delivered to the tumor, and our laboratory and others have shown that the limited blood supply may put tumors at a disadvantage in terms of drug delivery as compared to better-vascularized normal tissues (Hirst and Denekamp, 1979; Minchinton and Tannock, 2006; Tredan et al., 2007). Also, poor nutrition of tumor cells may lead to low rates of cell proliferation in some tumor regions (Hirst and Denekamp, 1979; Ljungkvist et al., 2002), and cells in such regions are likely to be resistant to cycle-active drugs as shown in Figure 1A.

#### Tumor acidity

The poor vascular organization and lack of lymphatic drainage of solid tumors contributes to a build up in metabolic byproducts such as lactic and carbonic acids leading to a reduced extracellular pH. The production of lactate arises from glycolysis – a favored route of energy production in tumors. Glycolysis typically takes place under hypoxic conditions, when oxidative phosphorylation is not possible, but in tumors glycolysis also takes place in oxygenated regions (Song et al., 2006). Tumor acidity influences drug uptake into tumor cells. When the extracellular tumor environment is acidic, chemotherapeutic drugs that are basic (such as doxorubicin, mitoxantrone, vincristine, and vinblastine) are protonated; this decreases cellular uptake since charged drugs pass through the cellular membrane less efficiently than those that are uncharged (Manallack, 2008). In contrast, drugs that are acidic (such as chlorambucil and cyclophosphamide) will tend to concentrate within cells. Even if basic drugs pass through the cellular membrane, sequestration within acidic organelles such as endosomes may occur, leaving less drug to attack tumor DNA and produce antitumor effects (Mayer et al., 1986).

#### Tumor hypoxia

Hypoxia is a hallmark of many different tumor types. The convoluted vasculature of tumors can result in insufficient oxygen supply through blood vessels as seen in Figure 1A. This type of hypoxia is known as chronic or diffusion limited hypoxia. Acute hypoxia may also occur in solid tumors due to intermittent blood flow.

Cells that reside far away from functional blood vessels may become hypoxic due to the limited diffusion of oxygen: the distance from blood vessels to hypoxic regions will depend on the rate of oxygen consumption by the tumor cells, but typically cells residing at a distance greater than 70 μm from functional blood vessels receive inadequate amounts of oxygen (Vaupel and Harrison, 2004). Hypoxic cells can be viable, but usually proliferate slowly, presumably due to their reduced production of ATP; however recent work from our laboratory has shown that as chemotherapy induces the death of cells close to blood vessels, hypoxic cells may reoxygenate and proliferate, presumably because of a better supply of nutrients and oxygen.

Hypoxia in tumors is associated with a poor clinical outcome as compared to patients with tumors lacking hypoxia (Hockel et al., 1996; Fyles et al., 2002; Nordsmark et al., 2005; Jubb et al., 2010). The presence of hypoxia leads to up-regulation of genes that promote a more malignant phenotype and favor cell survival. The transcription factor hypoxia inducible factor I (HIF-1) is induced, and causes the synthesis of angiogenesis-relevant proteins, suppression of apoptosis, and enhanced receptor tyrosine kinase signaling (Mizukami et al., 2007). These in turn favor epithelial to mesenchymal transition (EMT) – a process that is associated with tumor invasiveness and metastasis (Wilson and Hay, 2011). HIF-1 also induces the expression of carbonic anhydrase 9 (CA9) which favors the hydration of CO_2_ leading to the production of carbonic acid – further contributing to a decrease in extracellular pH (Potter and Harris, 2004).

Tumor hypoxia is linked with loss of the p53 tumor suppressor protein that may result in a loss of apoptotic ability (Haensgen et al., 2001). Furthermore, hypoxia confers radio-resistance because reactive oxygen radicals that are produced following radiation under well-oxygenated conditions contribute to DNA damage (Rofstad et al., 2000). Hypoxia may also inhibit the effects of chemotherapy via the same mechanism since in the presence of oxygen drugs such as doxorubicin can produce reactive oxygen species such as super-oxides that can damage DNA (Luanpitpong et al., 2012). Hypoxia has also been shown to down-regulate expression of DNA topoisomerase II, so that drugs such as doxorubicin and etoposide that target this protein will be inefficient (Ogiso et al., 2000).

Transient hypoxia can stimulate gene amplification, leading to increased expression of genes that encode proteins that cause drug resistance; these proteins include dihydrofolate reductase, with associated resistance to methotrexate and the multi-drug resistant transporter P-glycoprotein (Wartenberg et al., 2003). Increased expression of P-glycoprotein results in increased levels of substrate drugs being pumped out of cells thus resulting in inadequate intracellular levels to cause cytotoxicity (Matheny et al., 2001).

#### Factors influencing drug distribution within solid tumors

Anticancer drugs must reach target tumor cells through the vasculature. The penetration of drugs to tumor cells is reliant upon convection and/or diffusion. Convection depends on pressure gradients and given that the pressure within tumor blood vessels and the tumor interstitium are both quite high, there is probably minimal movement of drugs from the vasculature to the tumor via this mechanism (Kuszyk et al., 2001). Diffusion involves the movement of drugs along a concentration gradient, i.e., from areas where they are concentrated (within the vasculature) to less concentrated regions (the tumor interstitium). Larger molecules tend to move more slowly than smaller molecules via diffusion, and tissue penetration will depend on consumption by the cells (Tredan et al., 2007). Drugs that are water-soluble will diffuse more readily through the extracellular fluid, although the diffusion coefficient will depend on the nature of the ECM. Drugs with higher lipid solubility can penetrate into cells more easily (Undevia et al., 2005). Drug half-life is also an important determinant influencing drug penetration, since drugs with longer half-lives in the circulation have a better opportunity to establish themselves within tumor tissues (Undevia et al., 2005).

#### Quantifying drug distribution

Quantification of drug distribution is important in order to determine a drug’s ability to penetrate tissue within solid tumors. Both *in vitro* and *in vivo* techniques have been used for quantifying drug distribution. A common *in vitro* technique uses tumor spheroids, and adherent tumor cells can grow spheroids to up to 3 mm in diameter (Conger and Ziskin, 1983). Spheroids develop hypoxic areas as well as central necrosis once they have reached ∼500 μm in diameter (Vinci et al., 2011). Drug distribution in spheroids can be studied for fluorescent drugs, or by using autoradiography to determine the distribution of labeled drugs (Lesser et al., 1995; Kuh et al., 1999). An alternative is to generate multicellular layers (MCL) on collagen-coated micro-porous membranes: the rate of penetration can then be evaluated by adding a drug on one side of the MCL and measuring its concentration on the other as a function of time, as shown in Figure 1B (Wilson and Hay, 2011). Spheroids and MCL have been used to study the distribution of a wide range of drugs (Tannock et al., 2002), and most drugs show rather poor distribution in tumor tissue.

Drug distribution can also be studied in tumors grown in animals. Growth of tumors in window and ear chambers allows for direct observation of tumor microcirculation, but a disadvantage is that tumors are relatively small with limited areas of hypoxia and/or necrosis (Hak et al., 2010). Tissue sections can be obtained after drug treatment of animals bearing transplanted tumors or human tumor xenografts and used for immunohistochemical analysis. This analysis will allow the quantification of fluorescent drugs in relation to blood vessels or regions of hypoxia, and the technique can be applied to human biopsies (Lankelma et al., 1999; Primeau et al., 2005; Fung et al., 2012). These studies have revealed decreasing concentration of fluorescent doxorubicin, mitoxantrone, or topotecan with increasing distance from blood vessels (Hirst and Denekamp, 1979). Distribution of other drugs such as cetuximab, trastuzumab (Lee and Tannock, 2010), and melphalan (Saggar et al., 2013) within tumor sections can be quantified with the use of anti-IgG specific (for the former two) or melphalan DNA adduct specific (for the former) monoclonal antibodies that recognize the drug activity.

**Figure 2:**
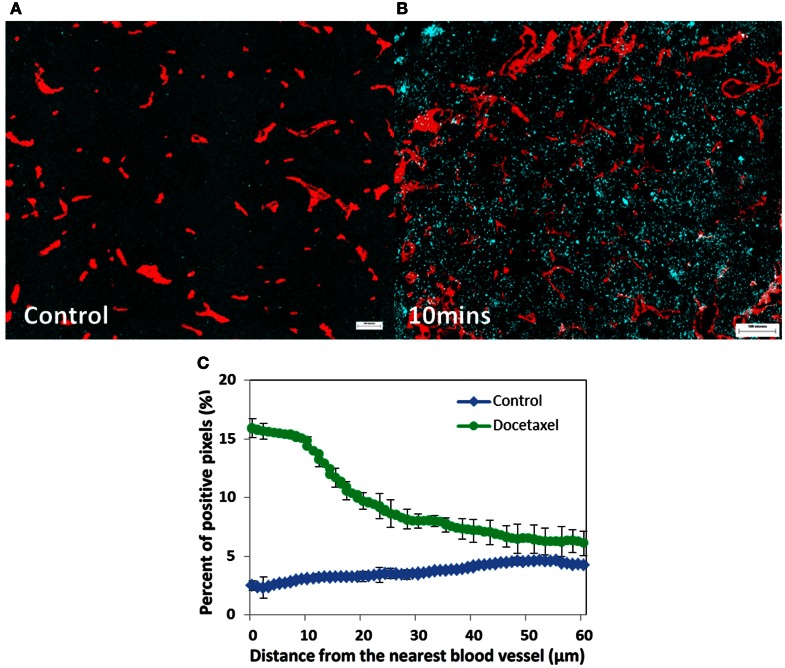
**Prostate cancer PC-3 xenografts (A) untreated control or (B) treated with docetaxel (15 mg/kg)**. **(A,B)** Show changes in γH2aX (in cyan), a biomarker of drug effect, in relation to tumor blood vessels (in red) at 10 min after injection. **(C)** Represents quantitative analysis of the distribution of γH2aX-positive cells in relation to the nearest blood vessel in tumors treated with docetaxel for 10 min (green line) and untreated controls (blue line). Points indicate average of six mice per group; bars, SE. ♢: control; ∘: docetaxel.

Most anticancer drugs are non-fluorescent so their distribution within tumor tissue is difficult to assess. An alternative is to evaluate molecular markers of drug effect, using antibodies that recognize cell proliferation (Ki67, cyclin D1, or bromodeoxyuridine incorporation into DNA), antibodies that mark cell death or apoptosis (e.g., caspase-3 or -6), and markers of DNA damage such as γH2aX. We recently used antibodies to γH2aX, caspase-3 or -6, and Ki67, and a computer-based algorithm, to quantify the distribution of (non-fluorescent) docetaxel (Saggar et al., 2013). Figure 2 depicts the expression of γH2aX following docetaxel treatment in xenografts; use of this and the other markers show that docetaxel also has limited distribution from tumor blood vessels.

Given the limited penetration of many chemotherapeutic agents, cells that are distal from blood vessels do not receive adequate amounts of drug to cause cell death. Thus, tumor cell repopulation arising from areas where cells are not killed and previously under-nourished (e.g., hypoxic regions) is probable, and indeed we have recently shown that previously hypoxic cells may reoxygenate and repopulate after treatment of human tumor xenografts with doxorubicin or docetaxel (Saggar et al., 2013).

#### Tumor autophagy

Autophagy is a cellular process of self-consumption characterized by sequestration of bulk cytoplasm, long-lived proteins, and cellular organelles into double-membrane vesicles called autophagosomes which are delivered to, and degraded in lysosomes (Larsson et al., 1985; Funderburk et al., 2010). The autophagosomal membrane requires a kinase complex consisting of class III phosphoinositol 3-kinase (PI3K), p150 myristylated protein kinase and Beclin1 (Atg 6). Subsequently, two further protein complexes are involved, the Atg4-Atg8 [also known as light chain (LC3/MAP1LC3B)] and the Atg12-Atg5/Atg7-Atg16 complex (Levine, 2007). Autophagy is thought to have at least three roles within the cell (Lee and Tannock, 2006; Levine, 2007): (1) it is a major pathway for quality control because it degrades damaged or superfluous cellular components in order to avoid mutational accumulation; (2) it may facilitate cell death as an alternative or complementary pathway to apoptosis; (3) it provides an alternative energy source by recycling cellular constituents during periods of metabolic stress to maintain cellular viability. Such stressors may include nutrient deprivation, hypoxia and cytotoxic agents, and markers of autophagy co-localize with hypoxia in tumor sections (Hoyer-Hansen and Jaattela, 2007). Hypoxic areas are reported to be primary sites of autophagy in 12 head and neck tumor cell lines (Rouschop et al., 2010) and recent data from our laboratory suggest that tumors grown from cells that do not express *Atg7* and *beclin-1* genes do not contain hypoxic regions.

Autophagy is prognostic of poor outcome in multiple tumor types, including cancers of the breast, lung, and colon (Karpathiou et al., 2011; Sivridis et al., 2011). High levels of autophagy have been associated with resistance to systemic therapy in several preclinical and clinical models presumably because it facilitates survival of stressed or damaged cells through recycling of cellular breakdown products (Yang et al., 2011). Hence targeting of autophagy with pharmacological agents may be a mechanism to improve the effectiveness of anticancer drugs for solid tumors.

### Strategies to improve therapy by modulating the tumor microenvironment

#### Inhibiting tumor autophagy

The Atg proteins are involved in autophagosome formation – a critical step required for autophagy to occur, therefore the inhibition of autophagy can be achieved by knockdown of *Atg* genes or by pharmacological inhibition. For example, deletion of *Atg7* and *Beclin1* inhibited autophagy induced by nutrient deprivation of cervical cancer cells and induced cell death (Yu et al., 2004) while stable knockdown of *Atg7* in human breast cancer cells inhibited cell growth in soft agar and tumor formation in nude mice (Kim et al., 2011). These strategies can also enhance tumor cell death induced by diverse anticancer drugs in preclinical models (Yang et al., 2011).

Agents which inhibit endosomal acidification, including (hydroxy)chloroquine and proton pump inhibitors (PPIs), can suppress autophagy and may therefore inhibit survival mechanisms for nutrient deprived cells (Marino et al., 2010). Luciani et al. (2004) reported the use of PPIs to sensitize cancer cells and solid tumors to various chemotherapeutic agents. Multiple mechanisms are probably involved, but appear to relate to changes in acidity in both intra and extracellular compartments of tumor cells. This group also reported that PPIs inhibit autophagy (Marino et al., 2010) probably because fusion of autophagosomes with acidic endosomes is central to the process, and we have confirmed this. Several studies have shown that PPIs such as omeprazole, esomeprazole, and pantoprazole have activity against human hematopoietic and solid tumors; they may revert chemo-resistance in drug-resistant tumors and directly induce killing of tumor cells (Yeo et al., 2004; De Milito et al., 2007, 2010). Growing evidence suggests that the major mechanism may be inhibition of autophagy.

#### Strategies to reduce interstitial fluid pressure

The interstitial fluid pressure (IFP) within solid tumors is often high (Heldin et al., 2004; Lunt et al., 2008), and this can inhibit the penetration of drugs into tumor tissue. This is particularly true in human pancreatic tumors that are extremely resistant to systemic cancer therapy (Olive et al., 2009; Provenzano et al., 2012). Raised IFP is due, at least in part, to a dense ECM and high cell density that lead to compression of blood vessels, and to inadequate lymphatic drainage (Ferretti et al., 2009). High IFP may have an adverse effect on treatment since it may cause vascular compression and inadequate drug delivery. A recent study by Provenzano et al. (2012) showed that there is an abundance of hyaluronic acid (HA) in the ECM of pancreatic tumors. HA is a large glycosaminoglycan that is associated with elevated IFP, and treatment with a HA-targeting enzyme (PEGPH20) was able to diminish HA levels and result in patent blood vessels and a corresponding increase in doxorubicin penetration (Provenzano et al., 2012). Other methods of improving vascular perfusion have also been investigated: Olive et al. (2009) reported that reduction in levels of tumor-associated stromal fibroblasts through disruption of Hedgehog signaling resulted in increased angiogenesis and greater penetration of gemcitabine into pancreatic tumors. The use of HA-targeting enzymes (PEGPH20) and Hedgehog signaling disruptors (GDC-0449 and LDE225) are being investigated in clinical trials.

#### Hypoxia-activated pro-drugs

Since hypoxic cells may survive after systemic drug treatment, and since tumor hypoxia confers a particularly metastatic and aggressive tumor phenotype, it is a logical target for new approaches to therapy. Hypoxia-activated pro-drugs (HAPS) have been developed, such that a pro-drug is administered in an inactive form, and is activated via a reduction reaction in hypoxic regions to damage DNA (Wilson and Hay, 2011). Since the pro-drug does not bind to DNA in oxygenated cells, it should diffuse readily to hypoxic tumor regions. Several HAPs have been investigated including tirapazamine, AQ4N, PR-104, and TH-302. Tirapazamine was investigated in phase III clinical trials, but due to limited clinical benefit (perhaps because of poor distribution in tumor tissue of both the pro-drug, and the activated drug), further clinical investigation was halted (Denny, 2010).

TH-302 is a 2-nitroimadazole whose nitro group undergoes fragmentation releasing the active bromo-isophosphoramide group that binds to DNA and causes cross-linkage to occur (Meng et al., 2012). TH-302 has been shown to decrease the hypoxic fraction and increase necrosis following treatment of many different tumors in animals (Sun et al., 2012). In a randomized phase II clinical trial of gemcitabine and TH-302 in pancreatic cancer, combined therapy increased progression-free survival from 3.6 to 5.6 months (Borad et al., 2012) and a phase III trial is in progress. Thus, TH-302 appears to be a promising addition to traditional chemotherapy, and recent studies in our laboratory suggest that it can inhibit the repopulation and reoxygenation of formerly hypoxic cells following treatment of human tumor xenografts with chemotherapy.

## Conclusion

Limited drug delivery to tumors is an important cause of treatment failure. The tumor microenvironment exerts effects that can alter the delivery of agents to neoplastic cells. Novel therapies that are able to leverage key characteristics of the tumor microenvironment such as hypoxia-activated pro-drugs and PPIs have potentials to result in improved therapeutic outcome.

## Conflict of Interest Statement

The authors declare that the research was conducted in the absence of any commercial or financial relationships that could be construed as a potential conflict of interest.
